# Metabolic profiling of glioblastoma and identification of G0S2 as a metabolic target

**DOI:** 10.3389/fonc.2025.1572040

**Published:** 2025-05-30

**Authors:** Jianlei Kang, Yujie Xu, Qitai Zhao, Ying Wang, Zhenyan He, Xin Xu

**Affiliations:** ^1^ Department of Neurosurgery, The Affiliated Cancer Hospital of Zhengzhou University and Henan Cancer Hospital, Zhengzhou, Henan, China; ^2^ Department of Oncology, Henan Provincial People’s Hospital, People’s Hospital of Zhengzhou University, People’s Hospital of Henan University, Zhengzhou, Henan, China; ^3^ Biotherapy Center and Cancer Center, First Affiliated Hospital of Zhengzhou University, Zhengzhou, Henan, China

**Keywords:** glioma, metabolic reprogramming, predictive model, GOS2, type I interferon

## Abstract

**Introduction:**

Metabolic reprogramming is a hallmark of cancer, yet its role in glioma remains poorly understood. Gliomas are characterized by a highly immunosuppressive tumor microenvironment (TME) and poor prognosis. This study systematically explores the relationship between glioma metabolomics, tumor phenotype, and the immune microenvironment.

**Methods:**

Bulk RNA sequencing data were retrieved from the Chinese Glioma Genome Atlas (CGGA) and The Cancer Genome Atlas (TCGA). Single-cell gene set enrichment analysis (ssGSEA) was employed to quantify seven nutrient metabolic pathways and immune infiltration. Consensus clustering was applied to group gliomas based on metabolic gene expression, and survival analysis was performed to evaluate survival differences across these clusters. A predictive model was constructed and validated using our cohort. Finally, we knocked out G0S2 in glioma cells and performed RNA sequencing to investigate differentially activated pathways. Additionally, *in vivo* experiments were conducted to explore the antitumor effects of G0S2 knockout in combination with PD-1 monoclonal antibody.

**Results:**

Significant metabolic differences were identified between low-grade gliomas (LGG) and glioblastomas (GBM), with consistent findings across both databases. We found that LGGs and GBMs exhibit distinct metabolic patterns. Consensus clustering revealed three metabolic subgroups, with the C3 subgroup demonstrating poor survival and enhanced infiltration of immunosuppressive cells. The predictive model showed robust performance in forecasting the survival of glioma patients. Functional analysis identified G0S2 as a key metabolic regulator highly expressed in gliomas. G0S2 knockout activated the type I interferon signaling pathway, enhanced CD8^+^ T cell functionality, and synergized with anti-PD-1 therapy, resulting in suppressed tumor growth and prolonged survival *in vivo*.

**Conclusion:**

These findings provide a comprehensive analysis of glioma metabolic patterns and identify G0S2 as a promising therapeutic target.

## 1 Introduction

Gliomas are the most common and difficult-to-treat tumors of the central nervous system. According to the World Health Organization (WHO) classification, gliomas are classified as WHO grade I gliomas, grades II and III diffuse gliomas based on molecular dysfunction and histopathology, or grade IV glioblastomas with many genomic alterations (1–3). Glioblastoma (GBM), the most aggressive primary brain tumor, demonstrated rapid progression and inevitable recurrence within 8–9 months post-diagnosis despite multimodal therapies encompassing surgical resection, radiotherapy, and alkylating chemotherapy (e.g.,temozolomide). The average survival time is only 18 months (4–7). Moreover, treatment options for recurrent glioblastoma are scarce, with second-line chemotherapy showing only modest activity against the tumor. Patients with recurrence usually survive for less than 10 months (8). Clinical trials on immunotherapy for glioma are underway and may benefit patients (9–11). Therapies against molecular targets that drive primary tumor growth have also been unsuccessful in clinical trials; therefore, new approaches are required (12).

Metabolic reprogramming is recognized as a hallmark of cancer and a key event in tumor progression and recurrence (13). Aerobic glycolysis (Warburg effect) is a metabolic feature of many solid tumors, including gliomas, and a flexible switch from aerobic glycolysis to mitochondrial metabolism enables tumor cell survival under stress (14). Glioma cells increase intracellular lipid, amino acid, and nucleotide stores through metabolic reprogramming (15). Mutations affecting isocitrate dehydrogenase (IDH) enzymes, which are components of the tricarboxylic acid (TCA) cycle, are prevalent in gliomas (16–18). Many studies have delineated the molecular circuitry linking specific genetic alterations to distinct metabolic phenotypes (19). Studies have discovered that IDH1 mutant glioma cells respond to medications that target enzymes in the *de novo* pyrimidine nucleotide synthesis pathway, providing new therapeutic options for patients with IDH mutations, which are common in gliomas (18). Researchers have observed high levels of amino acids, especially glycine and 2-aminoadipic acid, in grade IV gliomas, and N-acetyl aspartic acid in low-grade gliomas (2). Gliomas have also been shown to utilize enzymatic activity acquired through the common mutation in IDH to eliminate the migration of CD8^+^ T cells to tumors (20). Although cell metabolism is now recognized as a characteristic of gliomas, the relationship between systemic metabolomics, glioblastoma phenotype, and the immunological microenvironment has not yet been explored.

In this study, we compared the differences in seven major metabolic pathways between low-grade gliomas (LGG) and glioblastomas (GBM) as well as between primary and recurrent tumors. Additionally, we performed a subtype classification of gliomas based on their metabolic patterns. A survival prediction model was constructed using metabolism-related genes. Furthermore, we identified the metabolic gene G0S2 as a potential therapeutic target.

## 2 Materials and methods

### 2.1 Data acquisition and procession

Level 2 RNA-sequencing (RNA-seq) data and corresponding clinical information of patients with LGG and GBM from TCGA and CGGA were downloaded from their respective websites (TCGA: http://xena.ucsc.edu/; CGGA: http://www.cgga.org.cn/). Gene Expression Omnibus(GEO)data were downloaded from the GEO database (https://www.ncbi.nlm.nih.gov/geo/) with the accession number GSE43378. The metabolic genes were downloaded from a previous study (**Supplementary Table 1**) (12).

### 2.2 Estimation of score of metabolic process and immune cells in LGG and GBM

The R package “ssGSEA” was used to estimate the seven metabolic process using identified genes in glioma and GBM. The R packages “ssGSEA” and “MCP” were used to estimate the score of immune cells. “ssGSEA” could estimate 28 cell type of immune cells, and “MCP” could estimate 10 cell type of immune cells. The major parameters for ssGSEA were min.SZ=1,tau=1, ssgsea.norm=true. The R package “corrplot” were used to analyze and visualize correlation of metabolic process and immune infiltration using method of Spearman.

### 2.3 Consensus clustering and characterization of LGG and GBM

The R package “ConsensusClusterPlus” was used to cluster glioma and GBM using metabolic genes. The major parameters were reps=100, pItem=0.8, cluster method:hc, distance: euclidean. The expression of seven metabolic processes and differences in the clinical parameters in each cluster were visualized using the R package “pheatmap.” The R package “survival” was used to analyze the differences in overall survival (OS) among the three clusters. The R package “ggplot2” were used to reveal the difference in immune cells among three clusters using ANOVA test.

### 2.4 Construction and validation of prediction model

First, RNA-seq data of glioma (including LGG and GBM) from TCGA were randomly divided into training and test datasets at 1:1 ratio based on survival status. Subsequently, univariate analysis was performed to identify the metabolic genes that correlated with OS in the training datasets with threshold *p*<0.001. Next, the least absolute shrinkage and selection operator (LASSO) algorithm was used to select gene signatures that could predict OS in the training datasets. The maximum number of iterations was set to 1000 (maxit=1000) using 10-fold cross-validation. A multivariate Cox regression analysis was used to calculate the formula for the prediction model. The R packages “survival” and “survminer” were used to analyze the differences in OS between high- and low-risk groups in the test datasets, our clinical cohort, and GEO datasets. Furthermore, “survival ROC” was used to analyze receiver operating characteristic (ROC) curve between high- and low-risk groups in these datasets.

### 2.5 Reverse transcription PCR analysis

After cell collection, TRIzol reagent was added to extract total RNA, and the RNA concentration was measured using a Nanodrop spectrophotometer. A total of 1 µg of RNA was used for reverse transcription according to the manufacturer’s instructions (Vazyme Biotech Co., Ltd, #R333-01). The primer sequences were as follows: DPEP1, Forward: 5’-CAAGTGGCCGACCATCTGG-3’, Reverse: 5’-GGGACCCTTGGAACACCATC; G0S2: Forward: 5’-GGAAGGCTGGAACTCTACGA-3’, Reverse: 5’-TTCTTTGGAGCAGTCGGTGT-3’; and PLA2G2A: Forward: 5’-GAAAGGAAGCCGCACTCAGTT-3’, Reverse: 5’-CAGACGTTTGTAGCAACAGTCA-3’. Relative gene expression levels were normalized to those of GAPDH.

### 2.6 Identification of differently expressed metabolic genes between LGG and GBM

The R package “Limma” was used to identify DEMGs between gliomas and GBM. DEMGs were selected with threshold of adjust *p* value< 0.05 and log 2 fold change (Log FC)≥0.05. DEMGs were visualized using a volcano plot. Venn plots were used to show the intersecting genes that were upregulated and downregulated in GBM in TCGA and CGGA datasets.

### 2.7 Generation of G0S2 knock-out cell lines

Mouse glioma cell line GL261 was donated from Henan Key Laboratory of Brain Targeted Bio-nanomedicine, School of Life Sciences & School of Henan University, Kaifeng, China. Mouse G0S2-KO tumor cells (GL261) were generated using a lentiviral system. A small guide RNA (sgRNA) targeting G0S2 was designed using an online tool (https://sg.idtdna.com/site/order/designtool/index/CRISPR_CUSTOM), and its sequence used was 5’-GGCTGCACACCGTCTCAACT-3’. To package the lentivirus, HEK293T cells were co-transfected with relinked lentiCRISPR v2 (Addgene, catalog no. 52961), psPAX2 (Addgene, catalog no. 12260), and pMD2.G (Addgene, catalog no. 12259). The cells were incubated with filtered viral medium containing 6 mg/mL polyglutamine (Biosharp, catalog number: BL628A). Subsequently, puromycin selection was performed to obtain desired cells (0.5 mg/mL).

### 2.8 RNA sequencing analysis

After the tumor cells were collected, total RNA was extracted, and sequencing was performed using the MGISEQ-T7 platform according to the manufacturer’s instructions. Fragments Per Kilobase Million(FPKM)data were used for differential expression analysis, with the criteria set as log FC > 1 and *p* < 0.05. The clusterProfiler package was employed for KEGG and GO enrichment analyses, with significance thresholds of *p* < 0.05 and *q* < 0.05.

### 2.9 *In vivo* experiments

C57BL/6J mice aged 4–6 weeks were purchased from Charles River. GL261 and GL261-shG0S2 tumor cells were collected and counted. Subsequently, 1 × 10^6 tumor cells were subcutaneously implanted into the left inferior belly of mice. When the tumor volume reached approximately 50 mm³, mice were randomly allocated into groups and subsequently treated with either an interferon receptor inhibitor (MCE, #MAR15A3, 50 µg/mouse) or an anti-PD-1 monoclonal antibody (Selleck, BMS202, 200 µg/mouse) by tail vein or inhibitor of G0S2 (MCE, NS-3-008, 5mg/kg/mouse, ingest by mouth)(Every group N=5). The treatments were administered every two days for a total of three doses. The tumor volume was measured every two days, and the mice were monitored for vital signs. Mice were euthanized at the experimental endpoint or when subsequent measurements predict tumor volume exceeding 2000 mm³.

### 2.10 Flow cytometry analysis

After enzymatic digestion, the tumor tissues were processed into a single-cell suspension. The cells were then washed three times with Phosphate Buffered Saline (PBS) and stained with antibodies for flow cytometry, including FITC anti-mouse CD45 (BioLegend, #157213), PE anti-mouse CD8 (BioLegend, #140408), APC anti-mouse IFN-γ (BioLegend, #505810), and APC/Cyanine7 anti-mouse TNF-α (BioLegend, #506343). Staining was performed in the dark at 4°C for 15 minutes. Subsequently, the samples were analyzed using a BD flow cytometer.

### 2.11 Statistical analysis

Statistical analyses were performed using R software (version 3.6.3) and GraphPad Prism (version8.3.0). The Wilcoxon test was used to compare differences in metabolic processes and immune cell scores between the two groups. Analysis of variance was used to compare differences in immune scores among the three clusters. The log-rank test was used to compare the differences in OS among the three clusters or high- and low-risk groups. Two-tailed t-tests were used to compare differences between the two groups in the *in vitro* and *in vivo* experiments. Statistical significance was set at *p* < 0.05.

## 3 Results

### 3.1 Characterization of metabolic processes between LGG and GBM

**Figure 1:**
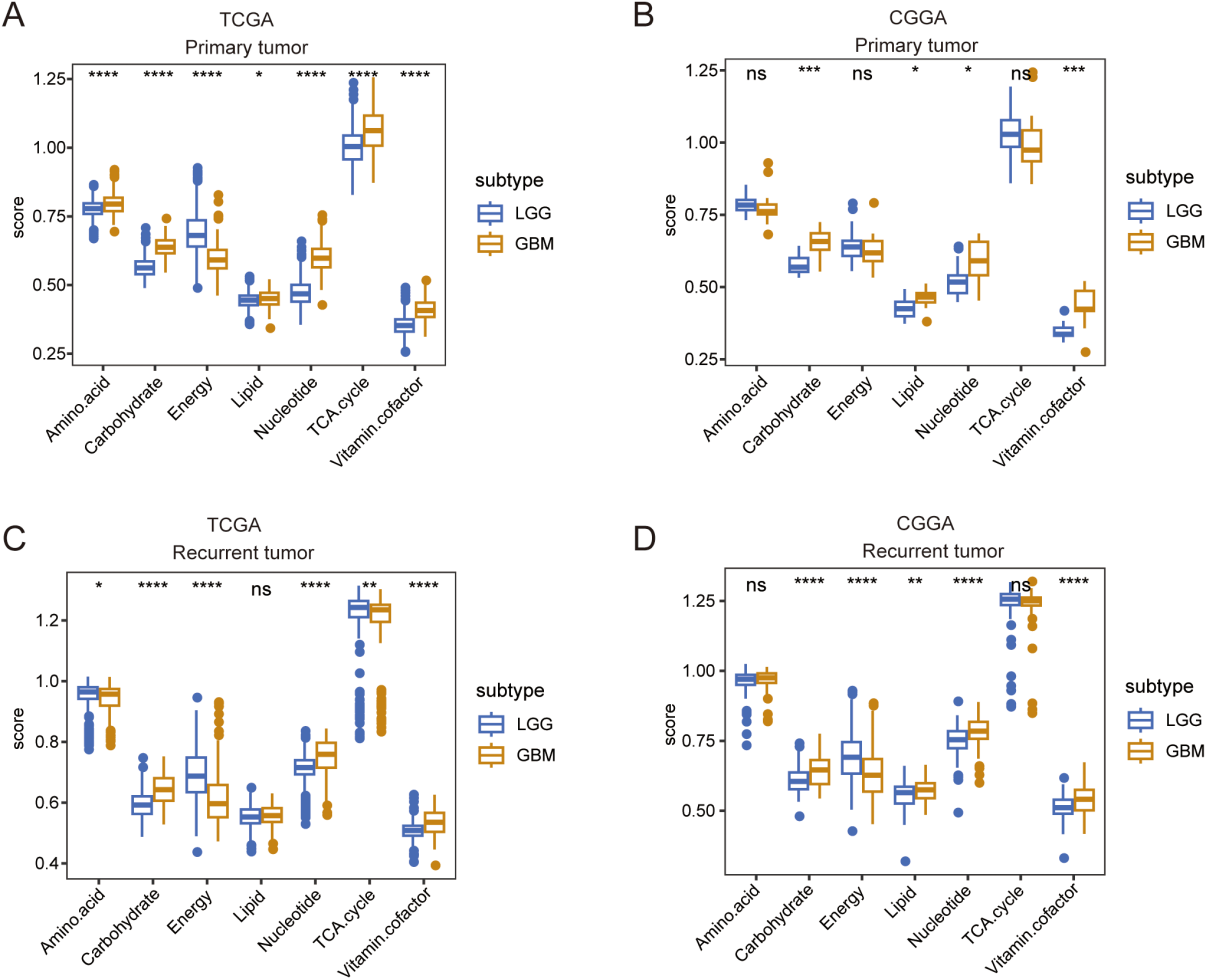
A comparison of seven major metabolic pathways between LGG and GBM. **(A, B)** Box plot showing the differences in seven major metabolic pathways between LGG and GBM in primary tumor tissue in TCGA and CGGA databases. **(C, D)** Box plot showing the differences in seven major metabolic pathways between LGG and GBM in recurrent tumor tissue in TCGA and CGGA databases. Wilcox.test ns, not significant, *p < 0.05, **p < 0.01, ***p < 0.001, ****p < 0.0001.

Given the different molecular patterns and clinical outcomes between LGG and GBM, we first explored alterations in the metabolic processes between LGG and GBM in both primary and recurrent tumor groups. In primary tumors, TCGA and CGGA analyses revealed an increased expression of carbohydrate, lipid, amino acid metabolism, TCA cycle, nucleotide, and vitamin cofactor metabolism in GBM, whereas amino acid metabolism and the TCA cycle were found to be more active in LGG, according to the CGGA database. (**Figures 1A, B**). In recurrent tumor tissues, we also observed that carbohydrate, lipid, nucleotide, and vitamin cofactor metabolism were more active in GBM. In contrast, TCGA and CGGA analysis revealed that TCA cycle were more active in LGG (**Figures 1C, D**). These findings reveal distinct metabolic patterns between LGG and GBM.

### 3.2 Dissecting the metabolic differences between primary and recurrent gliomas

**Figure 2:**
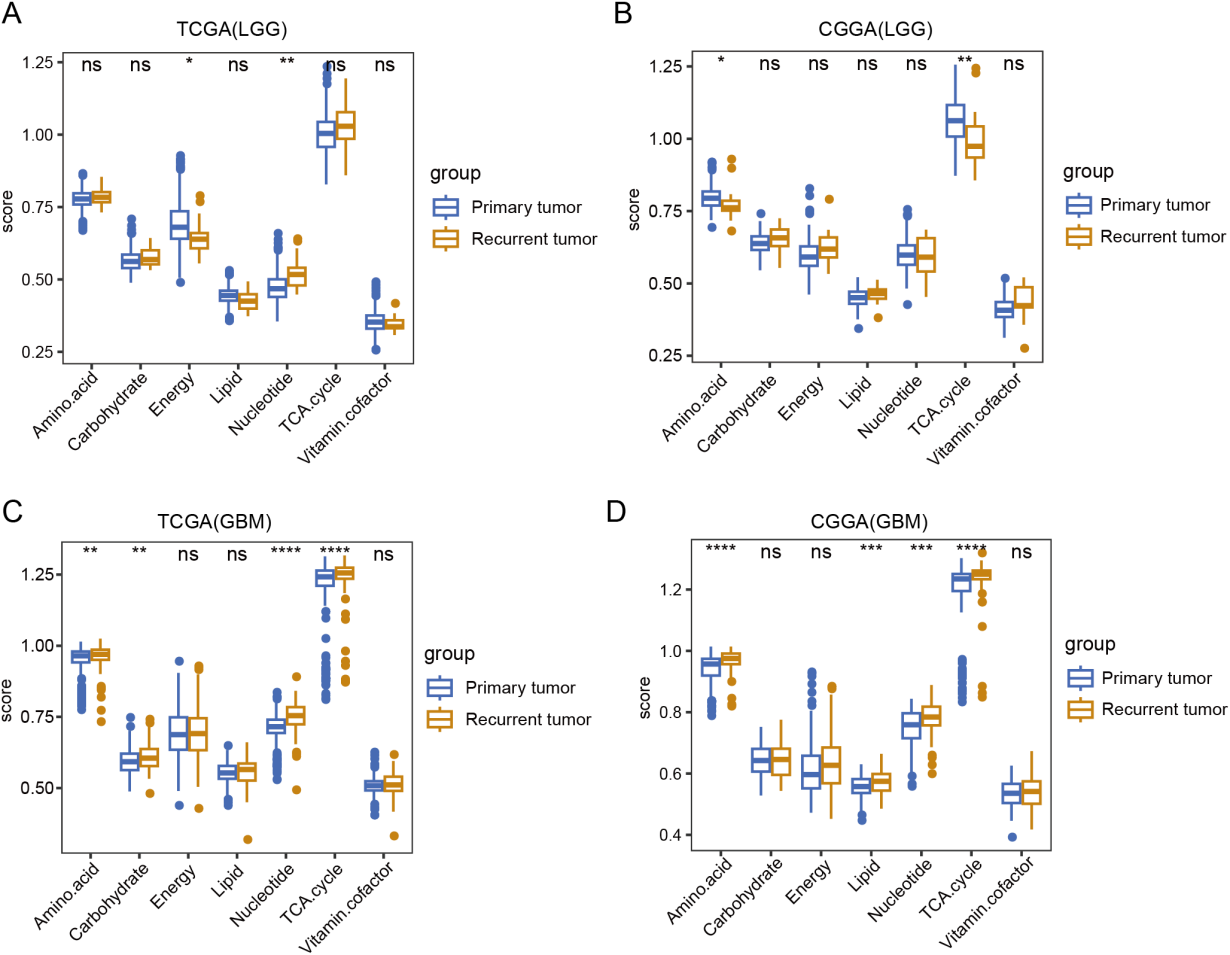
A comparison of seven major metabolic pathways between primary and recurrent tumor tissue. **(A, B)** Box plot showing the differences in seven major metabolic pathways between primary and recurrent tumor tissue in LGG in TCGA and CGGA database. **(C, D)** Box plot showing the differences in seven major metabolic pathways between primary and recurrent tumor tissue in GBM in TCGA and CGGA database. Wilcox.test, ns, not significant, * *p*<0.05, **p < 0.01, ***p < 0.001, ****p < 0.0001.

Next, we compared the metabolic processes between primary and recurrent tumors of LGG and GBM. TCGA data analysis revealed that in LGG, energy metabolism decreased, whereas nucleotide metabolism increased in recurrent tumors. Additionally, CGGA analysis indicated that amino acid metabolism and the TCA cycle were downregulated in recurrent tumors (**Figures 2A, B**). In GBM, both TCGA and CGGA analyses revealed that amino acid metabolism and the TCA cycle were upregulated in recurrent tumors, showing an opposite trend to that of LGG (**Figures 2C, D**).

### 3.3 Metabolic processes were correlated with immune infiltration

**Figure 3:**
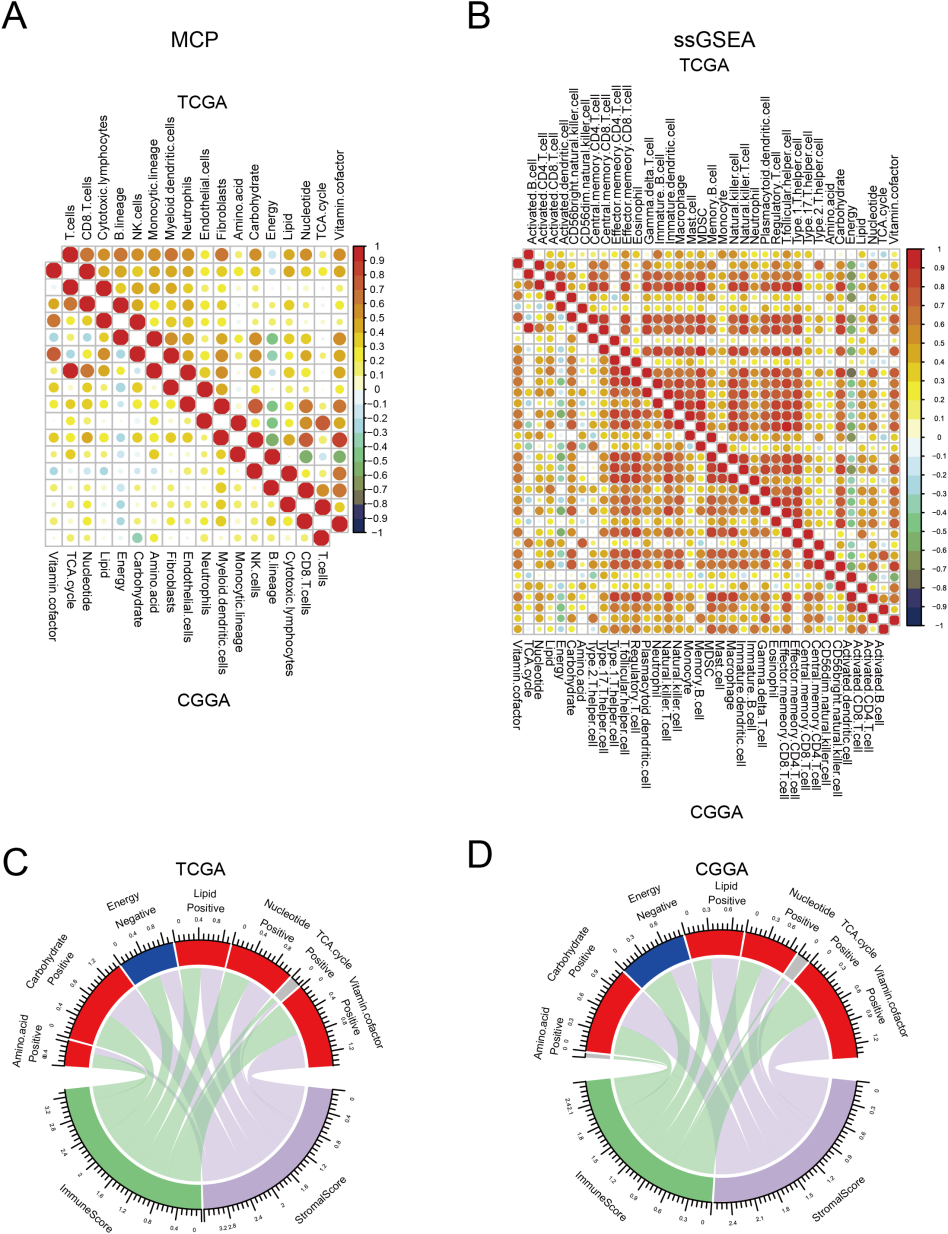
Correlation between seven major metabolic pathways and immune infiltration. **(A, B)** Heatmap showing the correlation between seven major metabolic pathways and immune cells in TCGA and CGGA database estimated by Microenvironment Cell Populations (MCP) and single-sample gene set enrichment analysis (ssGSEA). **(C, D)** Circle plot showing the correlation between seven major metabolic pathways and immune and stromal scores in TCGA and CGGA database.

The immune status in tumor tissues is associated with the natural progression of tumors and the outcomes of clinical treatment. Therefore, we evaluated the correlation between metabolic processes and immune cell infiltration. To this end, we first used “ssGSEA” and “MCP” methods to calculate the score of each cell type. We found that most immune cells had a strong correlation with each other in both TCGA and CGGA datasets, indicating a synergistic effect of these immune cells. Most metabolic processes positively correlated with immune infiltration, with energy and vitamin cofactors showing the strongest correlation. Notably, the energy process negatively correlated with other processes. In addition, we found that energy had a reverse correlation between immune cells in both TCGA and CGGA datasets (**Figures 3A, B**). Consistent with these results, both TCGA and CGGA analyses revealed that energy was negatively correlated with immune and stromal score (**Figures 3C, D**). These results suggest that alterations in metabolic processes shape immune infiltration in tumor tissues.

### 3.4 Classification of glioma based on metabolic processes

**Figure 4:**
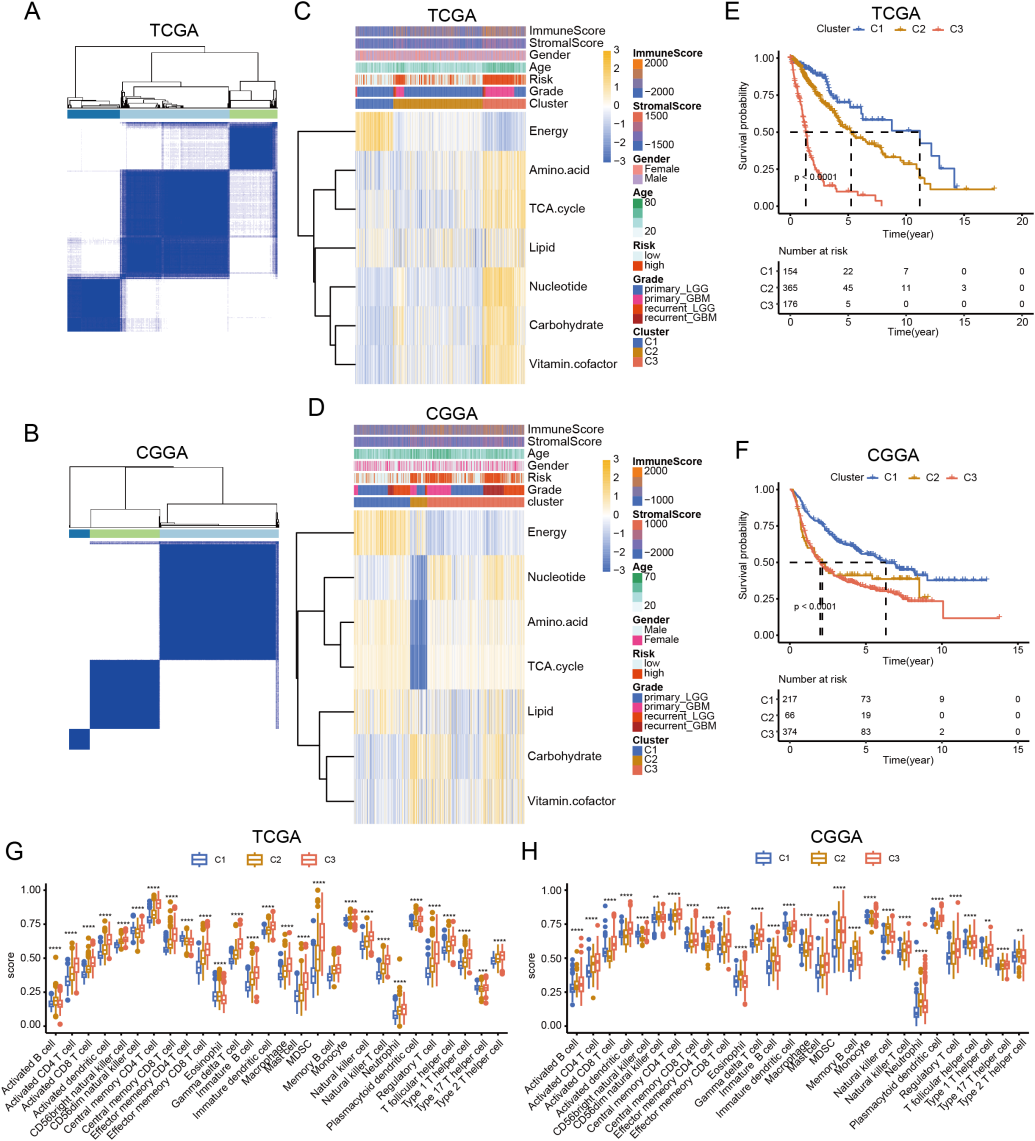
Classification of glioma based on metabolic processes. **(A, B)** Heatmap showing the clustering of glioma in TCGA and CGGA database. **(C, D)** Heatmap showing the expression of seven major metabolic pathways among three clusters in TCGA and CGGA database. **(E, F)** K-M curve showing the overall survival of three clusters in TCGA and CGGA database. **(G, H)** Box plot showing the level of immune cells in three clusters in TCGA and CGGA database. ANNOVA test, **p < 0.01, ***p < 0.001, ****p < 0.0001.

Metabolic reprogramming reflects tumor heterogeneity and is related to patient survival. Therefore, we performed a consensus clustering of gliomas based on these metabolic genes. Analysis of TCGA and CGGA datasets indicated that gliomas could be unsupervisedly clustered into three distinct subtypes. (**Figures 4A, B**). Although the number of samples in each cluster differed, the metabolic patterns were similar. In C1 cluster, energy was activated. C3 cluster was enriched in amino acids, carbohydrates, the TCA cycle, nucleotides, carbohydrates, and vitamin cofactor process. C2 cluster showed low expression of these metabolic process (**Figures 4C, D**). Survival analysis showed that patients in the C3 cluster had worse survival rates than those in the C1 or C2 clusters. In addition, C1 had the longest survival time among the three clusters, whereas the survival time of C2 was between those of C1 and C3 (**Figures 4E, F**). Further analysis revealed that C3 was enriched in most immune cells. Although some effector immune cells were abundant in C3, some immunosuppressive cells were also infiltrated in the C3 cluster, including myeloid-derived suppressor cells and regulatory T cells (**Figures 4G, H**). These results revealed that the metabolic classification of gliomas reflects the heterogeneity of the tumor.

### 3.5 Construction of a prediction model based on metabolic genes

**Figure 5:**
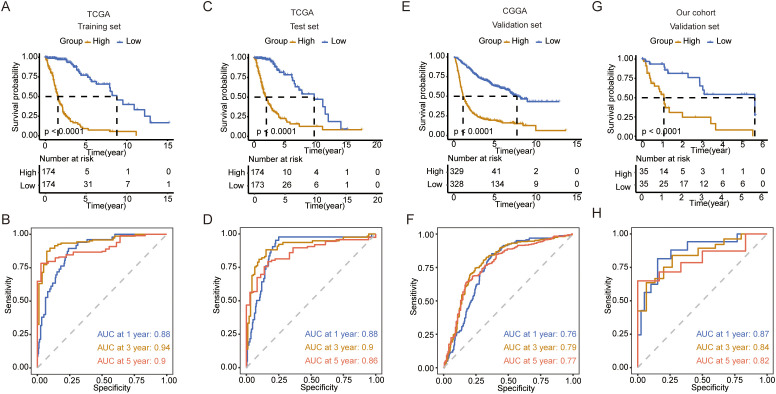
Construction and validation of the prediction model. **(A)** K-M curve showing the overall survival between the high- and low-risk groups in TCGA training set. **(B)** ROC curve showing the AUC in TCGA training set. **(C)** K-M curve showing the overall survival between the high- and low-risk groups in TCGA validation set. **(D)** ROC curve showing the AUC in TCGA validation set. **(E)** K-M curve showing the overall survival between the high- and low-risk groups in the CGGA validation set. **(F)** ROC curve showing the AUC in the CGGA validation set. **(G)** K-M curve showing the overall survival between the high- and low-risk groups in our cohort validation set. **(H)** ROC curve showing the AUC in our cohort validation set.

Next, we explored the functions of these metabolic genes, which influence the OS of patients with glioma. We first identified survival-related genes and used LASSO to optimize gene signatures (**Supplementary Figures 1A, B**). A seven-gene signature was selected and risk scores were calculated based on multivariate analysis (**Supplementary Figure 1C**). The prediction model performed well and could be clearly divided based on risk score (**Supplementary Figures 2A, B**). Five genes (TX1, MBOAT1, AOX1, HEXB, and GNG12) were upregulated in the high-risk group, and two genes (RPL3 and PIK3R1) were upregulated in the low-risk group in both TCGA and CGGA datasets (**Supplementary Figures 2C, D**). Next, we calculated the risk score for each patient in the training and test datasets. We found that patients with high risk scores had worse survival in both datasets (**Figures 5A, C**). ROC analysis revealed that the prediction model performed well on both datasets, with a high area under the curve (AUC) (**Figures 5B, D**). To further validate the prediction model, we used the CGGA, our cohort, and GEO to test its performance. The results indicate that the model accurately predicted patient survival within these datasets, demonstrating high sensitivity and specificity (**Figures 5E–H**; **Supplementary Figures 3A–D**).

### 3.6 Identification of G0S2 as a metabolic target to promote tumor progression

**Figure 6:**
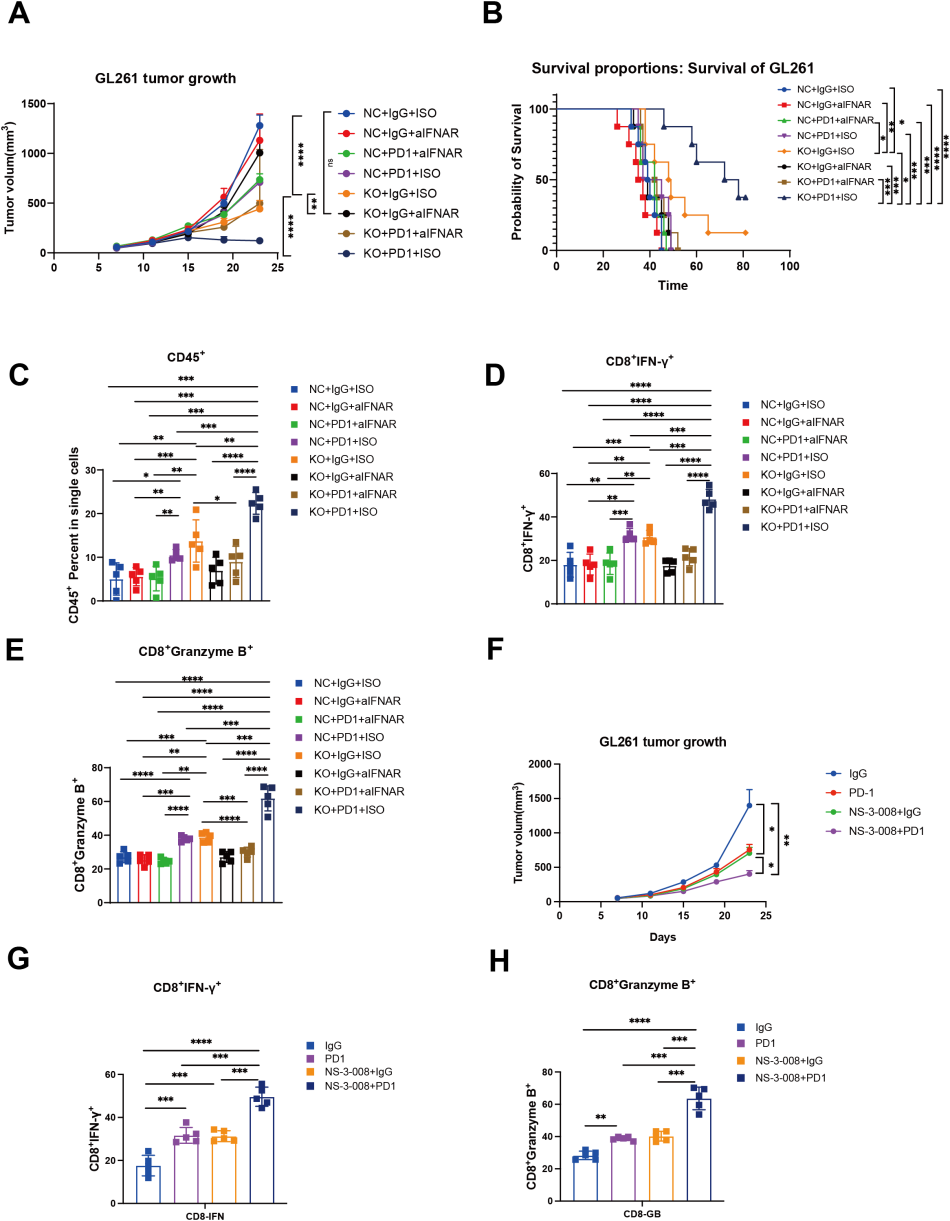
G0S2 knockout and G0S2 inhibitor inhibited tumor growth and promoted immune infiltration. **(A)** Tumor growth curve showing the tumor volume across these groups(Every group N=5). **(B)** K-M curve showing the survival across these groups(Every group N=5). **(C-E)** Bar plot showing the ratios of CD45^+^ cells, IFNγ^+^CD8^+^ T cells, and granzyme B^+^CD8^+^ T cells across these groups(Every group N=5). **(F)** Tumor growth curve showing the tumor volume in control, αPD-1,NS-3-008, and αPD-1combined NS-3–008 groups (Every group N=5) **(G, H)** Bar plot showing the ratios of IFNγ^+^CD8^+^ T cells and granzyme B^+^CD8^+^ T cells across these groups (Every group N=5). *t* test, *p < 0.05, **p < 0.01, ***p < 0.001, ****p < 0.0001.

To identify the potential metabolic target of glioma, we first analyzed the differentially expressed genes between tumor tissues and normal tissues in GBM and LGG, separately in primary and recurrent tumor types. The volcano plot showed dysregulated genes in these groups in both TCGA and CGGA datasets (**Supplementary Figures 4A–D**). By intersecting these genes, we identified three genes (PLA2G2A, DPEP1, and G0S2) that were upregulated in primary and recurrent tumor tissue (**Supplementary Figures 5A–D**). Further analysis using the online platform GEPIA revealed that these genes were associated with poor prognosis in patients with LGG (**Supplementary Figures 6A–C**). This finding was further validated by our own analysis of patient specimens, which confirmed elevated G0S2 expression in tumor tissues (**Supplementary Figure 6D**). Through comprehensive analysis of TCGA and CGGA datasets, we revealed that BCL2 as a G0S2-suppressed target gene that demonstrates low expression in glioblastoma (GBM) (**Supplementary Figures 7A, B**). Conversely, G0S2-activated target genes including CEBPA and PPARG were significantly upregulated in GBM. Notably, these differential expression patterns were not observed to be statistically significant in recurrent tumors, further supporting the above findings (**Supplementary Figures 7C–F**). To further validate the role of G0S2, we knocked out G0S2 in tumor cells (**Supplementary Figure 7G**). Enrichment analysis of differentially expressed genes between the knockout and control groups revealed that G0S2 knockout enhanced the response to type I interferons, while reducing oxidative phosphorylation (**Supplementary Figures 7H, I**). In the *in vivo* experiments, we treated two groups of mice with an IFNAR1 inhibitor and an anti-PD-1 monoclonal antibody. We observed that G0S2 knockout alone was sufficient to suppress tumor growth in mice, whereas administration of the IFNAR1 inhibitor in the control group promoted tumor growth. Notably, the combination of G0S2 knockout and anti-PD-1 antibody treatment significantly suppressed tumor volume and prolonged mouse survival (**Figures 6A, B**). Further analysis revealed that in the group with G0S2 knockout combined with anti-PD-1 antibody treatment, the proportion of CD45^+^ immune cells was significantly higher. Among these, the proportion of CD8^+^ T cells secreting IFN-γ and granzyme B was notably increased (**Figures 6C–E**). Further, the combination of G0S2 inhibitor (NS-3-008) and anti-PD-1 antibody treatment significantly suppressed tumor volume(**Figure 6F**) and promoting CD8^+^ T cells function (**Figures 6G, H**).These findings suggest that G0S2 may serve as a metabolic target that influences immune responses.

## 4 Discussion

Harnessing the clinical benefits of cancer metabolism requires defining the pathways that constrain cancer progression and understanding the background specificity of metabolic preferences and susceptibility in malignant tumor cells. Abnormalities in multiple metabolic pathways, such as glycolysis, amino acid metabolism, and lipid metabolism in glioma cells, meet the staggering energy demands in a hypoxic environment (21–23). Therefore, there is an urgent need to establish metabolic typing of gliomas and screen for therapeutic targets.

In this study, we conducted a comprehensive analysis of the differences in seven major metabolic pathways between LGG and GBM. Results demonstrated that carbohydrate, lipid, nucleotide, and vitamin cofactor metabolism were activated in GBM. Carbohydrate metabolism functions as a central axis, primarily ensuring a continuous energy supply, while serving as the origin for other metabolic and biosynthetic pathways, including amino acid and lipid metabolism (24). Abnormal tumor metabolism is often associated with tumor progression and drug resistance, and metabolic reprogramming of tumors is highly correlated with the heterogeneity of the tumor microenvironment (25). Hypoxia in the tumor microenvironment is a key driver of carbohydrate metabolism, primarily promoting glycolysis and lactate metabolism, which ultimately contributes to tumor progression (26–28). Carbohydrate metabolism serves as a central metabolic pathway that not only provides ATP for tumor cells but also influences nucleotide, amino acid, and lipid syntheses, ultimately shaping the overall metabolic profile of the tumor (29). Therefore, higher carbohydrate metabolism observed in GBM indicates a greater demand for energy, which is associated with a poorer prognosis. Consistent with these results, carbohydrate metabolism has been linked to poor survival in patients with glioma (30). Amino acid metabolism provides intermediate metabolites for the TCA cycle, thereby supporting energy production in tumor cells (31). Similarly, lipid metabolism plays a critical role in tumor cell division by contributing to the formation of cell membranes and the transmission of signaling pathways (32). However, why GBM, compared to LGG, activates these metabolic pathways remains a topic worthy of further investigation.

In the present study, we found that highly activated energy metabolism in tumor tissues was negatively correlated with immune cell infiltration. Energy metabolism primarily includes glycolysis and oxidative phosphorylation, among which glycolysis is more characteristic of the tumor microenvironment (33). In the tumor microenvironment, tumor cells primarily acquire energy through aerobic glycolysis, and the metabolic byproduct lactate significantly influences the immune composition and function of the microenvironment (34). Studies have reported that high concentrations of lactate can inhibit T cell proliferation and impair the maturation of dendritic cells (35, 36). Additionally, macrophages can take up lactate, which promotes their polarization into tumor-associated macrophages, leading to high expression of ARG1, which subsequently suppresses T-cell function (37). Interestingly, a positive correlation was observed between highly activated energy metabolism and patient survival.

In the present study, we identified G0S2 as a potential metabolic target. Numerous studies have reported that G0S2 is associated with tumor cell proliferation. G0S2 has been shown to promote anti-estrogenic and pro-migratory responses in breast cancer cells (38). Furthermore, G0S2 has been reported to be associated with lipid metabolism. It regulates the homeostatic proliferation of naive CD8^+^ T cells and inhibits oxidative phosphorylation in the mitochondria (39). KEGG enrichment analysis revealed that G0S2 knockout promoted activation of the type I interferon response. The type I interferon signaling pathway is a critical innate immune signaling pathway that regulates adaptive immunity. Recent studies have reported that this pathway is associated with anti-PD-1 monoclonal antibody therapy (40). In the *in vivo* experiments, we found that the combination of anti-PD-1 monoclonal antibody therapy and G0S2 knockout significantly inhibited tumor growth and extended the mouse survival of mice. We also found the combination of anti-PD-1 monoclonal antibody therapy and NS-3-008(G0S2 inhibitor) significantly inhibited tumor growth of mice and promoted the function of CD8^+^ T cells. These results suggest that G0S2 is not only associated with metabolism but may also play a critical role in regulating immune responses within the tumor microenvironment. While G0S2 inhibitors are currently being explored in chronic kidney disease (41), their potential applications in oncology remain unclear. Our study elucidates the therapeutic value of targeting G0S2 in tumor contexts.

## 5 Conclusion

In summary, this study comprehensively analyzed the expression patterns of seven major metabolic pathways in gliomas. Based on the distinct metabolic patterns, we identified three glioma subgroups with different survival outcomes and immune states. Furthermore, we developed a predictive model for the survival of patients with glioma using metabolic genes. Additionally, we found that G0S2 influenced the type I interferon signaling response in tumor cells and exhibited strong antitumor effects when combined with anti-PD-1 therapy. Our findings provide deeper insight into metabolic reprogramming in gliomas and suggest that G0S2 may serve as a potential therapeutic target.

## Data Availability

The datasets presented in this study can be found in online repositories. The names of the repository/repositories and accession number(s) can be found in the article/**Supplementary Material**.

## Supplementary material

Supplementary Figure 1 Gene identification for prediction model. **(A, B)** LASSO regression used for optimizing the gene signature. **(C)** Forest plot analysis showing the multivariate analysis of the gene.

Supplementary Figure 2 Construction of the prediction model. **(A, B)** Dot plot showing the survival status of patients with glioma in the high- and low-risk groups in TCGA training and validation set. **(C, D)** Heatmap showing the gene expression in the high- and low-risk groups in TCGA training and validation set.

Supplementary Figure 3 Validation of the prediction model. **(A, B)** K-M curve showing the overall survival between the high- and low-risk groups in TCGA glioma and GSE43378 sets. **(C, D)** ROC analysis showing the AUC in TCGA glioma and GSE43378 sets.

Supplementary Figure 4 Identification of differently expressed genes (DEGs). **(A, B)** Volcano plot showing the DEGs between tumor and normal tissue in primary tumor in TCGA and CGGA data sets. **(C, D)** Volcano plot showing the DEGs between tumor and normal tissue in recurrent tumor in TCGA and CGGA data sets.

Supplementary Figure 5 Identification of overlapping genes. **(A, B)** Intersection of genes upregulated in primary and recurrent tumor tissue in TCGA and CGGA database. **(C, D)** Intersection of genes downregulated in primary and recurrent tumor tissue in TCGA and CGGA database.

Supplementary Figure 6 Analysis expression of overlapping genes. **(A–C)** K-M curve showing the overall survival of patients with LGG and GBM grouped by DPEP1, G0S2, and PLA2G2A expression. **(D)** Dot plot showing the expression of DPEP1, G0S2, and PLA2G2A in tumor and adjacent tumor tissues. *t* test,**p* < 0.05.

Supplementary Figure 7 Identification of target genes and enrichment analysis of DEGs. **(A-F)** Box plot showing the expression of BCL2, CEBPA, PPARG between LGG and GBM in primary and recurrent tumor tissue in TCGA and CGGA databases (Wilcox.test). **(G)** Bar plot showing the gene expression of G0S2 between control and knockout group (*t* test). **(H, I)** Dot plot showing the upregulated and downregulated differently expressed genes between G0S2 knockout and control groups based on KEGG enrichment analysis. ns, not significant,**p* < 0.05, ***p* < 0.01, ****p* < 0.001, *****p* < 0.0001.
