# Supplementary figures and images for: Metabolic profiling of glioblastoma and identification of G0S2 as a metabolic target
Source: Front Oncol. 2025 May 30;15:1572040. doi: 10.3389/fonc.2025.1572040 (PMC12162274; doi:10.3389/fonc.2025.1572040)

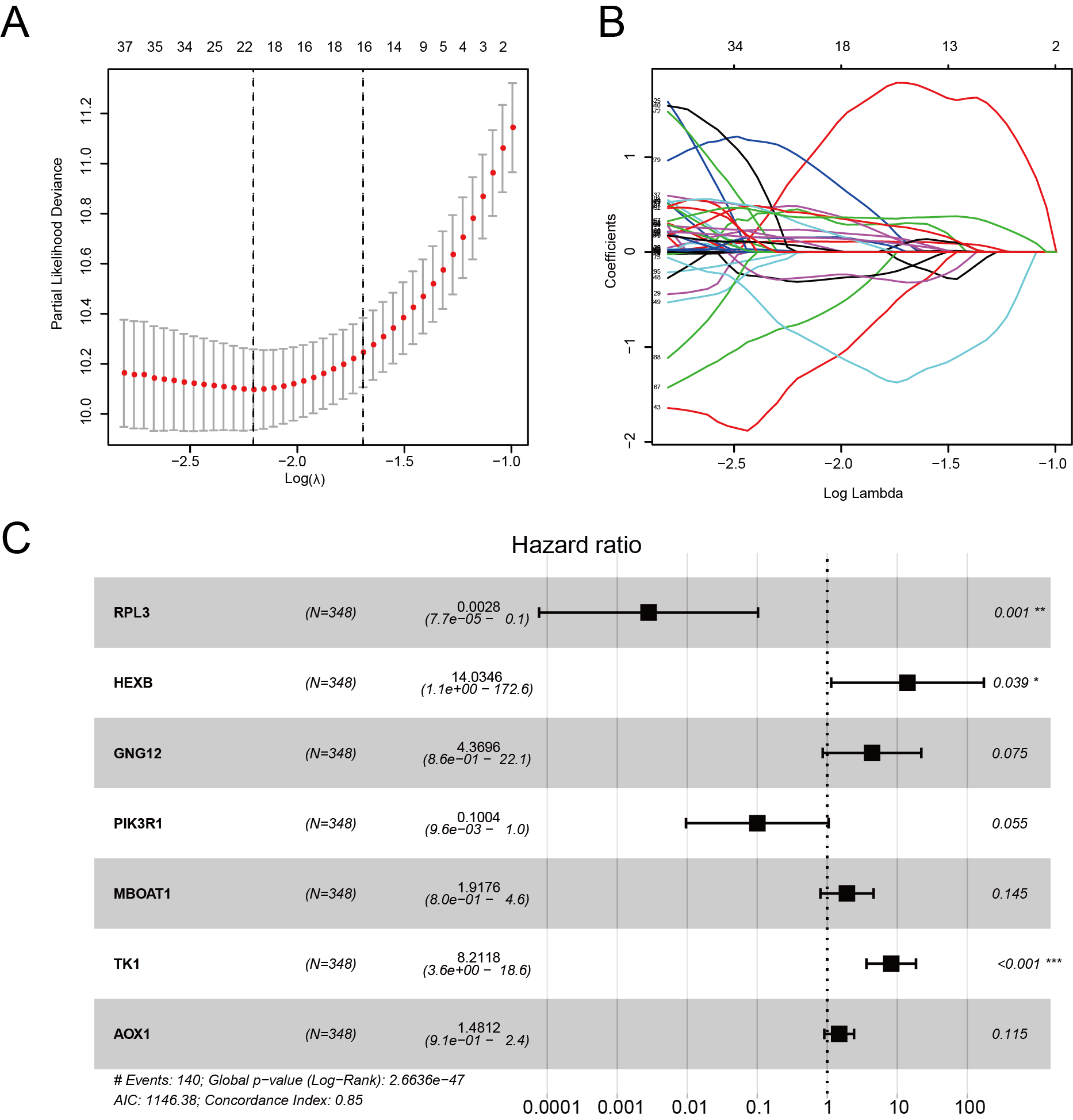

Supplement: Supplementary Figure 1 — Gene identification for prediction model. (A, B) LASSO regression used for optimizing the gene signature. (C) Forest plot analysis showing the multivariate analysis of the gene. [file Image1.tif]

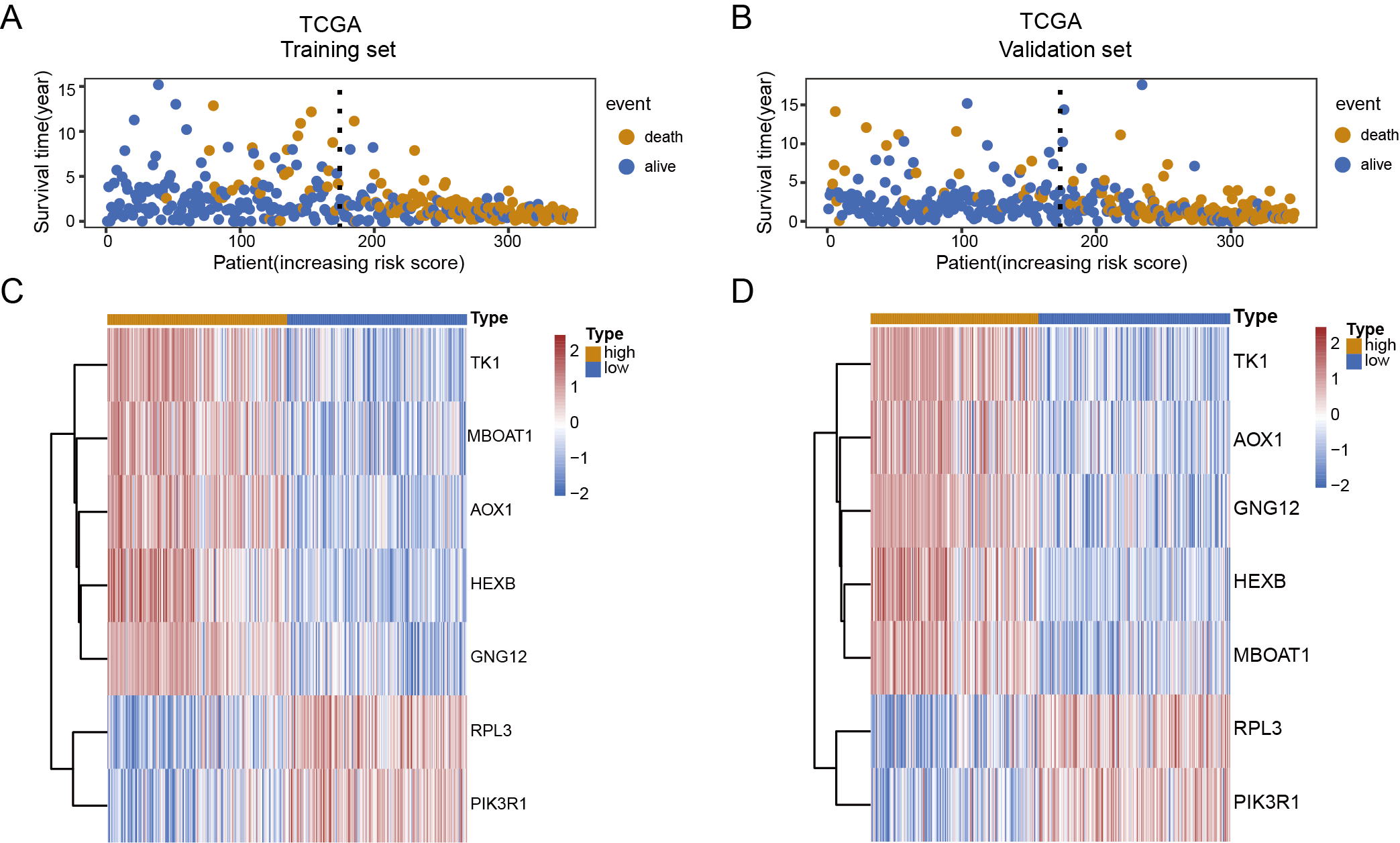

Supplement: Supplementary Figure 2 — Construction of the prediction model. (A, B) Dot plot showing the survival status of patients with glioma in the high- and low-risk groups in TCGA training and validation set. (C, D) Heatmap showing the gene expression in the high- and low-risk groups in TCGA training and validation set. [file Image2.tif]

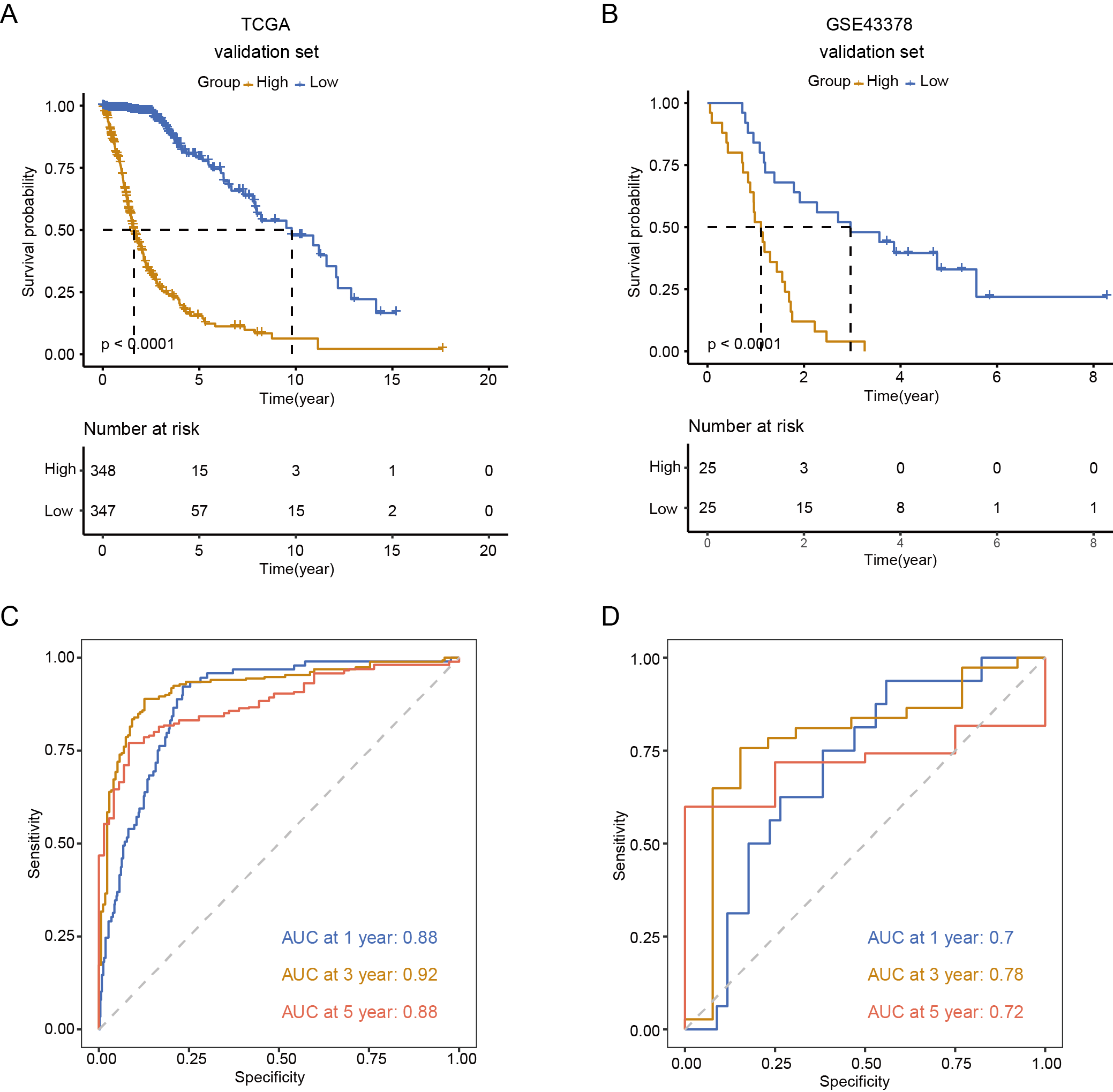

Supplement: Supplementary Figure 3 — Validation of the prediction model. (A, B) K-M curve showing the overall survival between the high- and low-risk groups in TCGA glioma and GSE43378 sets. (C, D) ROC analysis showing the AUC in TCGA glioma and GSE43378 sets. [file Image3.tif]

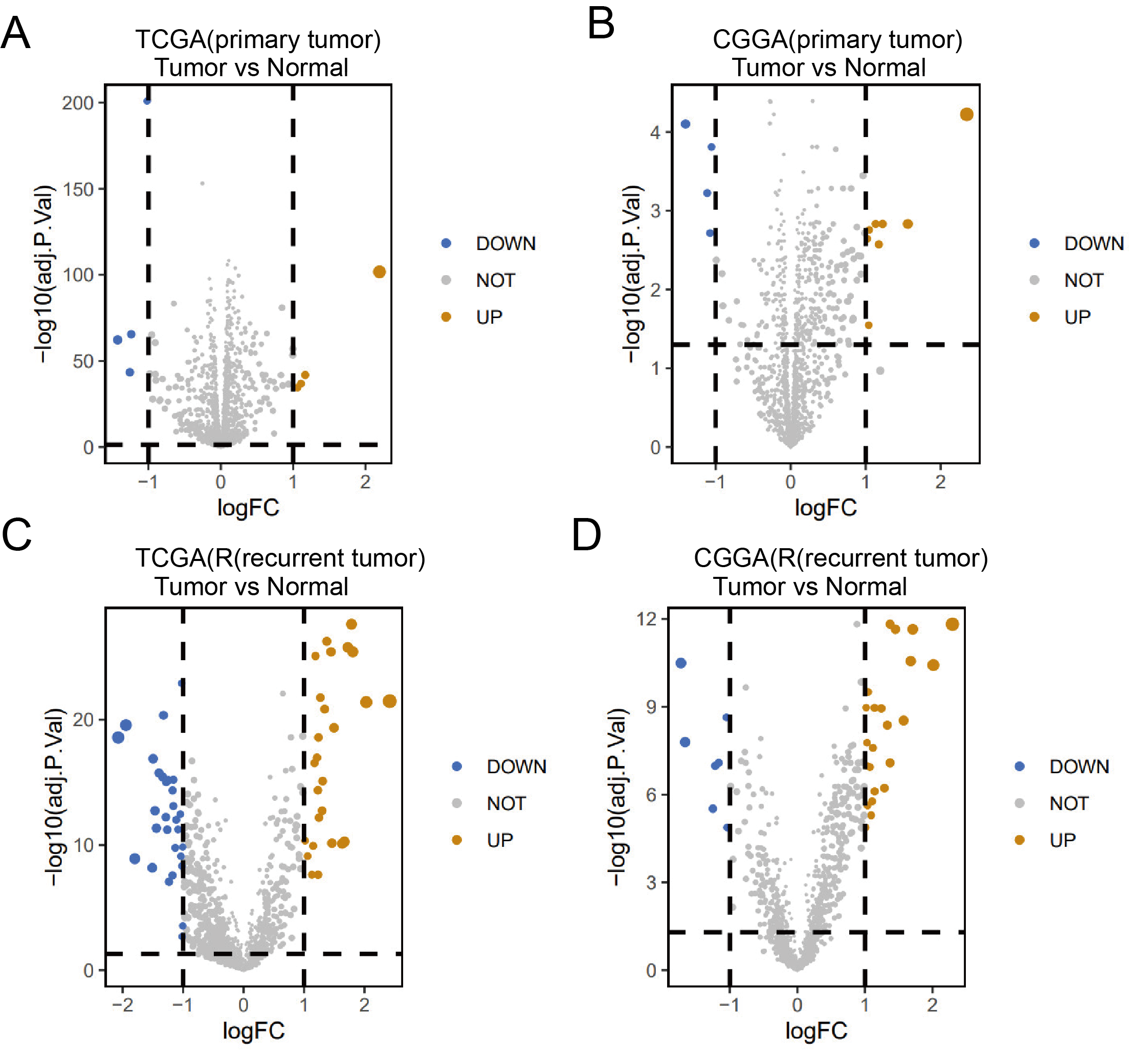

Supplement: Supplementary Figure 4 — Identification of differently expressed genes (DEGs). (A, B) Volcano plot showing the DEGs between tumor and normal tissue in primary tumor in TCGA and CGGA data sets. (C, D) Volcano plot showing the DEGs between tumor and normal tissue in recurrent tumor in TCGA and CGGA data sets. [file Image4.tif]

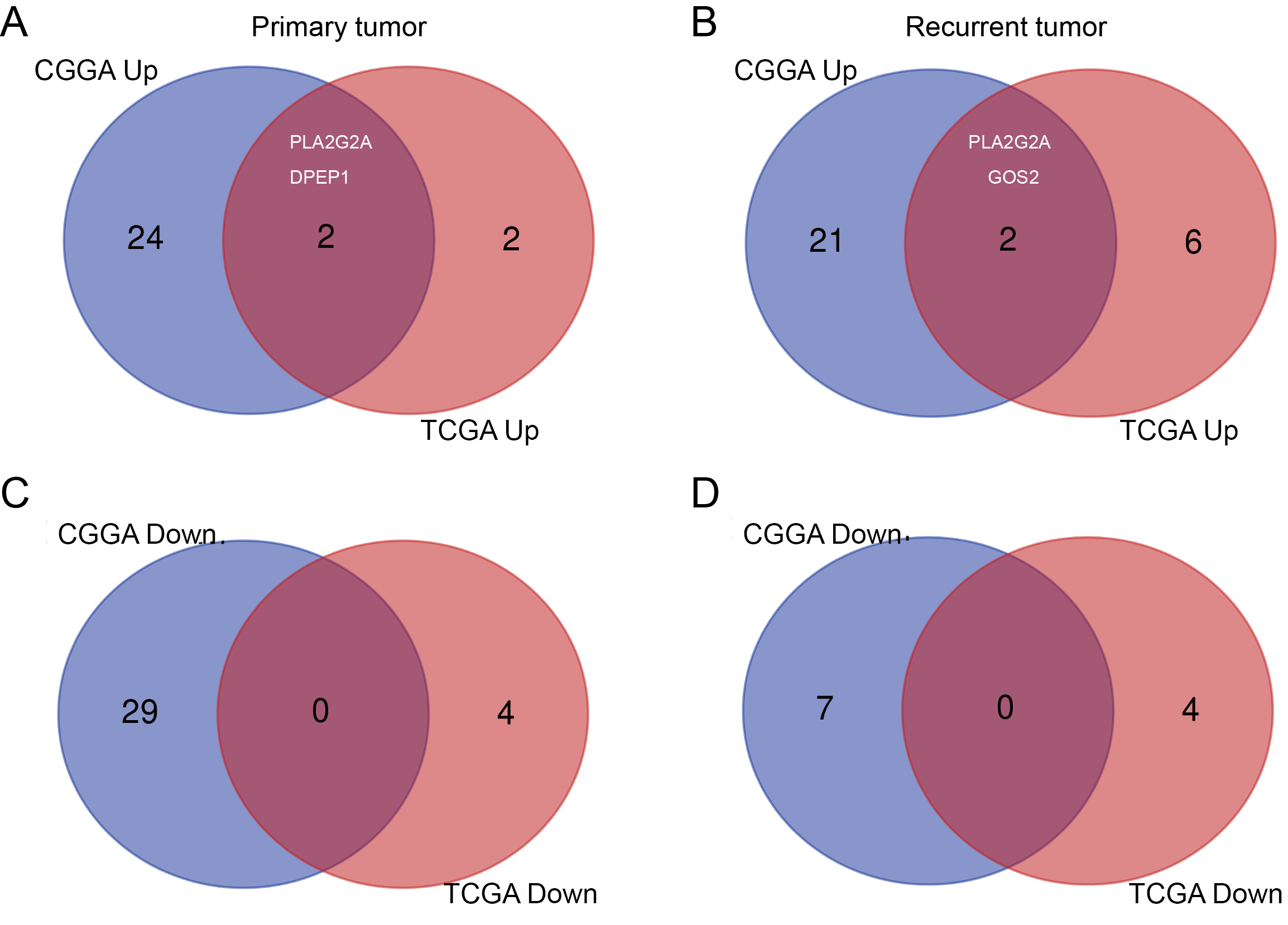

Supplement: Supplementary Figure 5 — Identification of overlapping genes. (A, B) Intersection of genes upregulated in primary and recurrent tumor tissue in TCGA and CGGA database. (C, D) Intersection of genes downregulated in primary and recurrent tumor tissue in TCGA and CGGA database. [file Image5.tif]

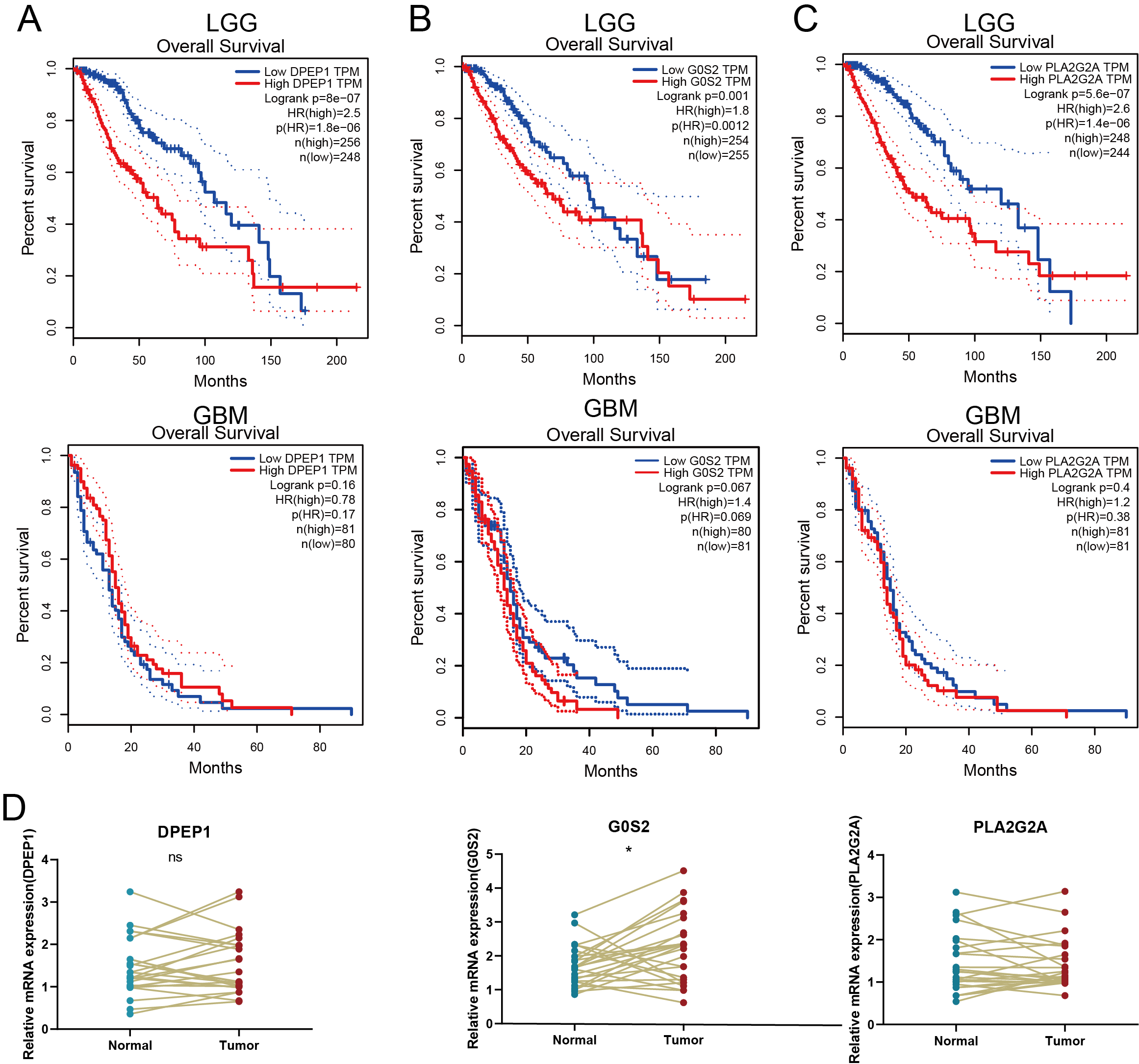

Supplement: Supplementary Figure 6 — Analysis expression of overlapping genes. (A–C) K-M curve showing the overall survival of patients with LGG and GBM grouped by DPEP1, G0S2, and PLA2G2A expression. (D) Dot plot showing the expression of DPEP1, G0S2, and PLA2G2A in tumor and adjacent tumor tissues. t test,*p < 0.05. [file Image6.tif]

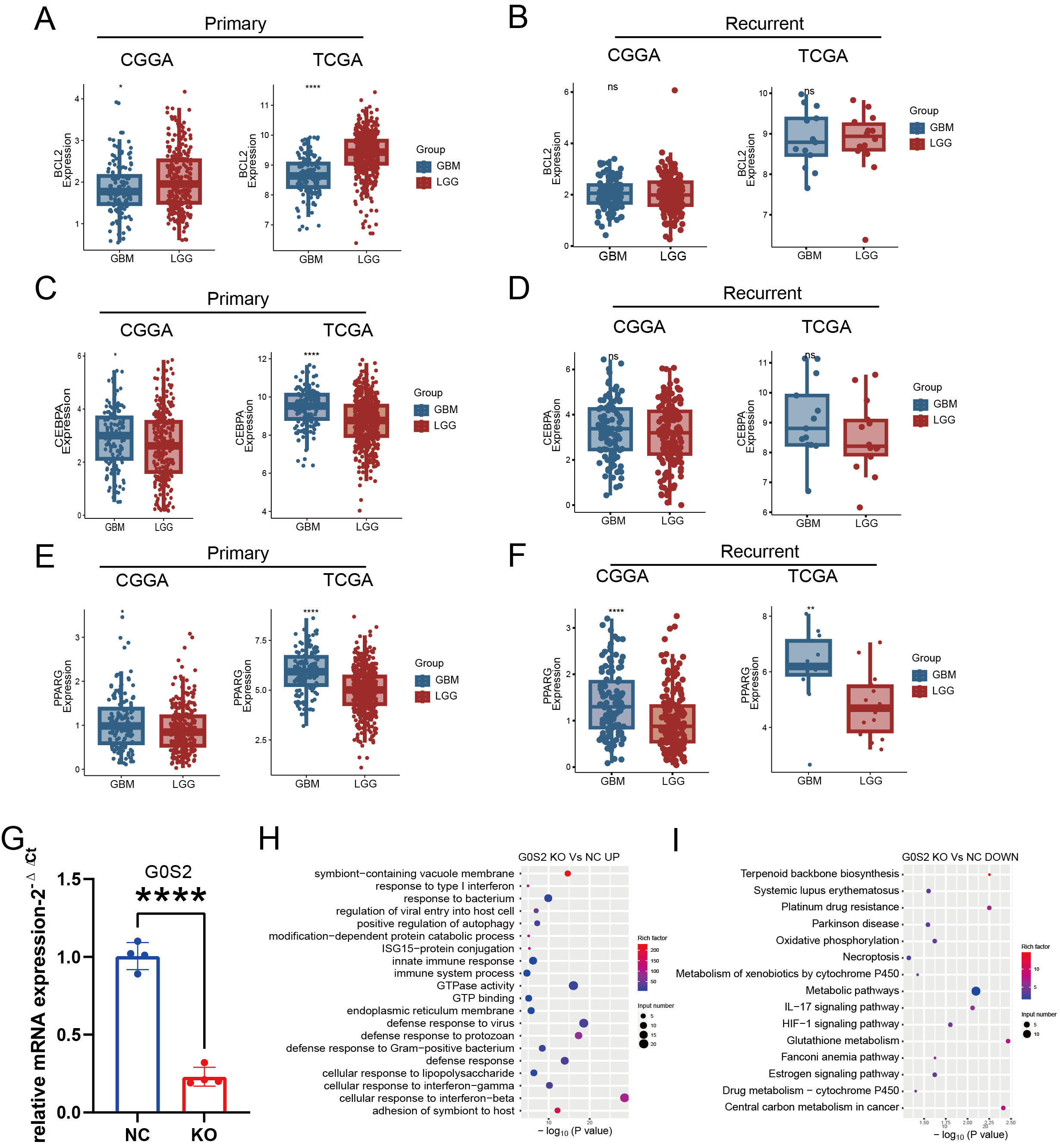

Supplement: Supplementary Figure 7 — Identification of target genes and enrichment analysis of DEGs. (A-F) Box plot showing the expression of BCL2, CEBPA, PPARG between LGG and GBM in primary and recurrent tumor tissue in TCGA and CGGA databases (Wilcox.test). (G) Bar plot showing the gene expression of G0S2 between control and knockout group (t test). (H, I) Dot plot showing the upregulated and downregulated differently expressed genes between G0S2 knockout and control groups based on KEGG enrichment analysis. ns, not significant,*p < 0.05, **p < 0.01, ***p < 0.001, ****p < 0.0001. [file Image7.tif]
